# Recruiting Student Health Coaches to Improve Digital Blood Pressure Management: Randomized Controlled Pilot Study

**DOI:** 10.2196/13637

**Published:** 2020-08-25

**Authors:** Elena Vasti, Mark J Pletcher

**Affiliations:** 1 University of California, San Francisco School of Medicine Stanford, CA United States; 2 Department of Biostatistics and Epidemiology University of California, San Francisco San Francisco, CA United States

**Keywords:** mobile health, hypertension, coaching, health-related behavior, mobile phone

## Abstract

**Background:**

Hypertension is a significant problem in the United States, affecting 1 in 3 adults aged above 18 years and is associated with a higher risk for cardiovascular disease and stroke. The prevalence of hypertension has increased in medically underserved areas (MUAs). Mobile health technologies, such as digital self-monitoring devices, have been shown to improve the management of chronic health conditions. However, patients from MUAs have reduced access to these devices because of limited resources and low health literacy. Health coaches and peer training programs are a potentially cost-effective solution for the shortage of physicians available to manage hypertension in MUAs. Activating young people as student health coaches (SHCs) is a promising strategy to improve community health.

**Objective:**

This pilot study aims to assess (1) the feasibility of training high school students as health technology coaches in MUAs and (2) whether the addition of SHCs to digital home monitoring improves the frequency of self-monitoring and overall blood pressure (BP) control.

**Methods:**

In total, 15 high school students completed 3-day health coach training. Patients who had a documented diagnosis of hypertension were randomly assigned to 1 of the 3 intervention arms. The QardioArm alone (Q) group was provided a QardioArm cuff only for convenience. The SHC alone (S) group was instructed to meet with a health coach for 30 min once a week for 5 weeks to create action plans for reducing BP. The student+QardioArm (S+Q) group received both interventions.

**Results:**

Participants (n=27) were randomly assigned to 3 groups in a ratio of 9:9:9. All 15 students completed training, of which 40% (6/15) of students completed all the 5 meetings with their assigned patient. Barriers to feasibility included transportation and patient response drop-off at the end of the study. Overall, 92% (11/12) of the students rated their experience as very good or higher and 69% (9/13) reported that this experience made them more likely to go into the medical field. There was a statistically significant difference in the frequency of cuff use (S+Q vs Q groups: 37 vs 17; P<.001). Participants in the S+Q group reported better BP control after the intervention compared with the other groups. The average BP at the end of the intervention was 145/84 (SD 9/18) mm Hg, 150/85 (SD 18/12) mm Hg, and 128/69 (SD 20/14) mm Hg in the Q, S, and S+Q groups, respectively.

**Conclusions:**

This pilot study demonstrates the feasibility of pairing technology with young student coaches, although challenges existed. The S+Q group used their cuff more than the Q group. Patients were more engaged in the S+Q group, reporting higher satisfaction with their SHC and better control of their BP.

## Introduction

### Background

Despite advances in management, hypertension remains a significant public health challenge in the United States, affecting approximately 33% of adults aged above 18 years [1,2]. Hypertension is associated with a significantly higher risk for cardiovascular disease and stroke [3]. From 2004 to 2014, the death rate directly attributable to hypertension increased by 7.6% and the total number of deaths from hypertension increased by 34% [3]. Factors such as race and socioeconomic status (SES) are major social determinants that influence an individual’s risk of hypertension. In one meta-analysis, lower occupational status and level of education were associated with odds ratios for hypertension of 1.31 and 2.02, respectively [4]. Another prospective trial demonstrated that incident hypertension was lower in participants from higher SES groups than in lower SES groups, suggesting that having limited social and economic resources plays a role in the disproportionate burden of hypertension seen in disadvantaged neighborhoods [5].

Mobile health (mHealth) offers a unique opportunity for improving blood pressure (BP) management [6,7]. Patient motivation, medication adherence, and diastolic BP have all been shown to improve with access to digital BP monitoring devices [8,9]. In rural areas, mHealth has been shown to improve outcomes. One study [10] demonstrated that the implementation of mHealth strategies, such as smart messaging systems, in rural health care centers across Lebanon significantly improved mean systolic BP in comparison with controls. These interventions have also been shown to improve adherence to BP medication regimens [11]. However, many of these studies require intensive resources to carry out mHealth interventions, including dedicated field researchers or community health workers. Although effective in the short term, it can be challenging to allocate the appropriate resources to these interventions. One qualitative study in rural Uganda discovered that providers and health care workers both articulated that a lack of patient education, limitations in time with patients, and funding for patient education were substantial barriers to implementing successful programs [12]. Health literacy, which was defined by Nielsen-Bohlman in 2004 as “the degree to which individuals have the capacity to obtain, process and understand basic health information and the services needed to make appropriate health decisions,” is an important variable to consider in the intervention of health programs. Improving the health literacy of patients allows them to become agents of change in their own health trajectory, which can reduce the burden on the health care system. Lower health literacy is often associated with patients from underrepresented and vulnerable groups and can contribute to widening health disparities in chronic diseases, such as hypertension [13]. Health literacy is also related to mHealth adoption. It is strongly associated with patients’ perceived ease of use and perceived usefulness of digital devices [14]. Thus, patients who are arguably the most likely to benefit from mHealth interventions potentially face barriers related to health literacy that limit their access to them. Health coaching has become an increasingly used strategy in low-income settings to manage BP outside of the clinic [15]. One study that examined health coaching as a possible strategy to prevent rehospitalization for chronic obstructive pulmonary disease (COPD) exacerbation randomized 215 patients hospitalized for COPD to either health coaching sessions with written action plans for exacerbations or usual care. The investigators trained volunteers as health coaches and evaluated the rate of COPD-related hospitalizations. There was a statistically significant difference of up to 9 months after discharge, and the investigators concluded that this is a potentially feasible approach for patients with chronic diseases that predispose them to hospitalization [16]. In addition, peer health coaching, where nonmedical community members are trained as health coaches, has been shown to be cost-effective and efficacious [17]. Social epidemiologists have begun to target youth civic engagement (YCE) as a strategy to promote community health in a social context [18], but to our knowledge, the impact of YCE *directly* on patient care has not been explored. In addition, bringing vibrant, young people into the health coaching intersection with digital technology is also an evidence-free zone. iPads have increasingly become a part of secondary education and have shown enhanced creativity and increased collaboration between students and teachers [19]. Our team felt that high school students’ exposure to technology in schools would uniquely qualify them as health coaches at the intersection of mHealth and health coaching. Using our understanding of the benefits of health coaching and mHealth in reducing the burden of hypertension, the focus of this study was to demonstrate that these 2 entities could synergistically improve BP monitoring and health behaviors.

### Objectives

This pilot study investigated an innovative approach for implementing a smartphone-based BP monitoring program in a medically underserved area (MUA) by engaging young people. First, we hypothesized that high school students could be trained as student health coaches (SHCs) to assist patients with smartphone devices for BP management. Second, we asked the question, “does the addition of weekly SHC visits to the use of digital BP monitoring devices improve patient engagement and overall BP control?”

## Methods

### Recruitment of Participants and Students

Participants aged above 18 years with a documented diagnosis of hypertension were recruited from a rural primary care clinic in Stockton, San Joaquin County, California, from June to July 2016. As recruitment took place at a small clinic with the primary care physician (PCP) available, patients who did not have chart documentation of hypertension but were verified by the PCP to be prescribed antihypertensive agents or currently attempting lifestyle changes for hypertension were included. This clinic was selected because it was the only primary care clinic located in South Stockton, San Joaquin County, California, where health disparities are most prevalent. All participants needed to own or have access to a Bluetooth-enabled smartphone. Patients were excluded if they were diagnosed with hypertension secondary to renal or endocrine disorders.

SHCs aged between 14 and 18 years were selected from a charter school in South Stockton, San Joaquin County, California. This charter school was a health academy, where the students who were enrolled had expressed an interest in careers in health care. The principal was actively involved in the development of this program as an extracurricular activity for students during the summer to build their resumes and skillsets. The University of California, San Francisco (UCSF) Institutional Review Board (IRB), who approved the protocol, required SHCs to complete research credentials. However, they did not require parental consent for participation, as the research activities were taking place in public places, such as the clinic itself or a coffee shop, if it was more convenient for the SHC. This was deemed to be of minimal risk for students in secondary school. They completed an extensive 3-day training on health coaching techniques. The curriculum included motivational interviewing; *ask-tell-ask* strategies for identifying barriers to patients' adherence to BP management; creating specific, measurable, achievable, realistic, and time-based (SMART) goals; and identifying hypertensive emergencies. SHCs were given a binder with reference sheets for use during their meetings. One day of the training was focused on using the QardioArm device itself. The health coaching curriculum was provided by the UCSF Center for Excellence in Primary Care. SHCs were all required to update an electronic tracking spreadsheet with deidentified notes from their meetings with patients. The purpose of the tracking spreadsheet was to ensure that SHCs were effective at guiding patients to create an action plan in the first visit and to document how they guided their patients through challenges and provided encouragement. Real-time feedback was provided by the research coordinator (EV) to the SHCs to improve the quality of the coaching sessions.

### Study Design

Participants were randomized in a 1:1:1 ratio to 1 of the following 3 groups: *SHC+QardioArm (S+Q)*, *QardioArm alone (Q)*, or *SHC alone (S)*. Randomization was open, and a computerized randomization algorithm was used to assign patients to 1 of the 3 groups. We planned to recruit 30 participants. Each patient who met the inclusion criteria was given information on the trial and was assigned to an intervention arm after they signed a consent form. We followed this system so that participation would not be influenced by group assignment and, therefore, there would be minimal differences between the groups. The 5-week intervention consisted of (1) weekly meetings with the SHC (S+Q and S arms) and (2) provision of a QardioArm BP home monitoring device to be used at the patient’s discretion (S+Q and S arms).

Qardio Inc, a digital monitoring device company based in San Francisco, California, donated QardioArm BP devices, which uploaded data to the patient’s smartphone. Patients were enrolled concurrently in the UCSF Health eHeart (HeH) study, and the HeH technical team carried out BP data collection. This study was approved by the UCSF IRB.

### Data Collection and Analysis

This pilot study used a mixed methods approach. SHCs completed postintervention surveys, which included both quantitative and qualitative data. Differences in pre- and postintervention BP across all 3 groups were reported. The frequency of QardioArm use and the number of active QardioArm users between the S+Q and Q groups were analyzed with a two-sided *t* test. Qualitative data reported by patients are quoted directly.

## Results

A total of 27 eligible participants were enrolled in the study. The baseline characteristics are displayed for 89% (24/27) of the participants (Table 1). The remaining 3 participants never returned to the baseline surveys.

**Table 1:** Baseline characteristics of Health eHeart participants in the San Joaquin County, California, cohort (N=24).

Group		QardioArm alone	Student health coach alone	S+Q^a^
Number of respondents^b^, n (%)		8 (89)	8 (89)	8 (89)
Age (years), mean (SD)		55 (15)	55 (6)	58 (17)
**Race and ethnicity, n (%)**				
	Black	2 (25)	3 (37.5)	3 (37.5)
	White	0 (0)	1 (12.5)	3 (37.5)
	Asian	2 (25)	0 (0)	0 (0)
	Hispanic	0 (0)	0 (0)	0 (0)
	Other Pacific Islander	1 (12.5)	0 (0)	1 (12.5)
	No answer	3 (37.5)	4 (50)	1 (12.5)
**Health insurance, n (%)**				
	MediCal/MediCare	3 (37.5)	3 (37.5)	3 (37.5)
	Health Plan of San Joaquin	2 (25)	1 (12.5)	2 (25)
	Blue Cross	0 (0)	1 (12.5)	0 (0)
	Care First	0 (0)	0 (0)	1 (12.5)
	Alignment	0 (0)	0 (0)	1 (12.5)
	None	3 (37.5)	3 (37.7)	1 (12.5)

^a^S+Q: student+QardioArm.

^b^One participant from each intervention arm did not return a baseline survey.

### Feasibility of High School Students as Health Coaches

All 15 SHCs completed training and at least one meeting to create a lifestyle plan with their assigned patient. Patients were assigned to SHCs in a nonrandom way at an orientation event held at the clinic where SHCs introduced themselves to patients and signed up for the 5 meetings together. Any of the participants or SHCs who could not attend the orientation event were individually contacted by the study coordinator and assigned patients based on the patient’s and SHC’s availability. One hypertensive emergency was accurately identified by an SHC, who followed the protocol and advised the patient to go to the emergency room for treatment. Follow-up surveys were completed by 80% (12/15) of the students. These SHCs rated their overall experience favorably, with 92% (11/12) of the SHCs reporting their experience from very good (8) to exceptional (10) on a 10-point scale. When asked how this project influenced their future plans to go into the medical field, 69% (9/13) of SHCs reported that they were more likely to go into the medical field. Notably, all (10/10) the SHCs who were assigned to patients randomized into the S+Q group reported that the feedback from the QardioArm device enhanced patient motivation and improved BP management. SHCs noted that they enjoyed the autonomy of working with patients the most. For example:

I absolutely loved the fact that this project allowed me to become an actual health provider. It was like I was her doctor and I’m sure it will prepare me for the future.

JT, 17 years

I enjoyed meeting with patients and actually being involved with tackling health disparities.

MS, 17 years

Of the 15 SHCs, 6 (40%) completed all 5 meetings, with 5 (33%) of these SHCs assigned to the S+Q group. SHCs reported difficulty with patient retention in the program:

...the patients would sign up and not show up, and it was pretty sad for the mentors, because some of us were actually looking forward to meeting them.

SN, 15 years

My two patients specifically stopped coming to meet up with me after a few days.

JT

In addition, SHCs kept weekly logs of their meeting notes on a spreadsheet and submitted them to the research coordinator during each week of the intervention (Table 2). The SHCs were all able to create effective SMART goals with patients, as shown in the sample of two of the SHC responses in the table.

**Table 2:** Example of student health coach–logged visit summaries for student+QardioArm and student health coach–alone groups.

Week of intervention	S+Q^a^	Student health coach alone
Week 1	Health Goals made: Meditation videos on YouTube 3 times a week for 30 min a time. Goal is to reduce stress levels. Has Bell’s Palsy and feels like she gets some eye pain when she gets anxious.	Patient made action plan goal to exercise 3 days a week for an hour. He plans on exercises Mondays, Wednesdays, and Fridays by walking around Victory Park or by going to the gym. Patient started diet about 2 weeks before starting the study. His diet includes having smoothies for breakfast as well as oatmeal. He has also cut carbohydrates, starches, and unhealthy sugars from his diet. Since he is already on a diet, he made another action plan goal to continue his diet to eat healthier.
Week 2	The meeting was a success. She completed and over exceeded her health Goal and even wanted to walk for 10 min, once a week, alone without her dog. He vision is still blurry because of her Bell’s Palsy, but she has arranged an eye doctor’s appointment. Meeting dates have been rearranged also.	Patient feels very confident with his action plan. He has exercise from Monday-Friday in the mornings for an hour, which is more than his original [*sic*]^b^ action plan states. Patient has maintained his diet and is sleeping better. Pateint [*sic*] has also lost 10 pounds and has set his own goal to lose weight. His goal weight is 170 pounds. The patient overall wants to be healthier and not have to take medication.
Week 3	In this meeting, my patient decided to add another health goal. Beside being already fantastic they wanted to cut down on the slts [*sic*]. We talked a little bit about her stress, and she was mentioning how her mother may contribute a little to her stress. She deffinately [*sic*] said that Bell’s Palsy was one of her major problems that stress her out, but she also said she’s been feeling a lot [*sic*] better. She had question about the program and what was going to happen after all five meeting were over and what we would do? She also experienced some technical issues with her Quardio [*sic*] Arm monitor, so I assured her I would ask. She also wnated to know what would happen with the Quardio [*sic*] arm after the five visits, and if she would return it? Beside her adding another [*sic*] health goal my patient has beeing [*sic*] following up with her other previous expectations and says she’s been doing great, and that she certaianatly [*sic*] has seen a differece [*sic*] in her overall life style.	No show
Week 4	even though she ran a little late because of a traffic jam on the freeway, she was an excellent patient and came. She’s been cutting down on her stress levels by listening to the meditation sound tracks, and she’s been walking for 30 min with her grandchildren, and besides that she has also been cutting down on the salts. She also confesses she likes eating chips, so she will stay away from those too. I explained to her after the five sessions we could meet up outside [*sic*] the clinic but it was totally up to her. I also let her know to add any concern about the Quardio [*sic*] arm and its performance on the feedback sheet that will hopefully be given to her by me. She will also be keeping the Quardio [*sic*] Arm after her participation in the case study. But she wanted to know if other patients were experiencing anything wrong with their Quardio [*sic*] Arm? she also said she’s pretty proud of her reading and significantly reduced them. Although she is a little scepticle [*sic*] because she has another High Blood Pressure monitor for her wrist and says that the wrist one, shows a different reading than the Quardio [*sic*] Arm.	Patient has been experiencing any problems and been doing well with his action plan. Patient was cleared to receive [*sic*] CPAP^c^ machine. Patient stated that he feels great, better than ever. Patient has been getting better sleep and has more energy. Patient as exercised for the past week Monday-Friday walking at Victory Park for an hour in the mornings and has been keeping up with his diet.
Week 5	No show	Patient appeared to be happier compared to the first meeting. Patient says he lost another 9 pounds. His current weight is 220 pounds, he started at 240. The patient feels good. He was been keeping up with his diet and exercising. He plans to keep these up in order to reach his goal weight, 170 pounds. He states that his energy has improved. He exercises for 5 days a for 1 hour and 30 min at a time. He has stated that the chanlleges [*sic*] he has faced include not want to exercises, wanting to get unhealthy foods, and keeping everything consistent [*sic*]. He was able to not give into these challenges and has benefited from it. Patient and I said our farewells.

^a^S+Q: student+QardioArm.

^b^SHC responses reported exactly as written in the originally logged visit summaries and are not edited for spelling or grammar to preserve the primary data.

^c^CPAP: Continuous Positive Airway Pressure.

### Patient Outcomes

**Figure 1:**
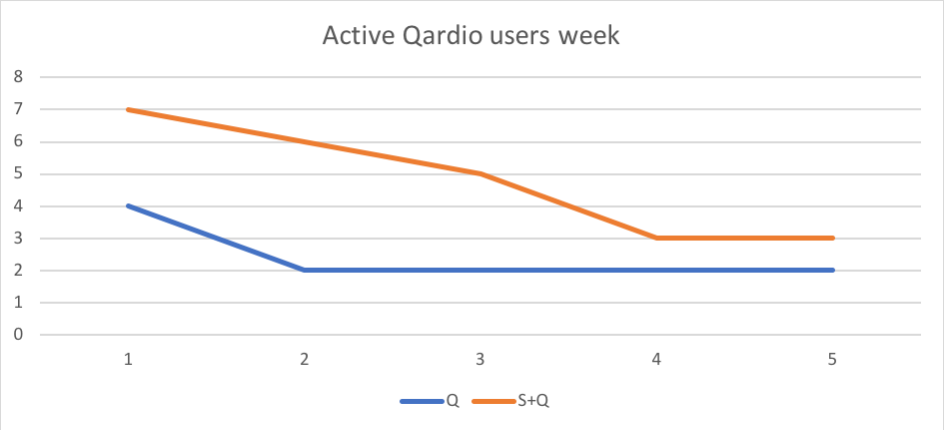
Active number of QardioArm users/week.

There was a total of 97 and 264 distinct uses of QardioArm in the Q group and S+Q group, respectively. We found a statistically significant difference in the average total number of uses of the BP monitor during the 5-week intervention (average total number of uses for S+Q vs Q groups: 37 vs 17; *P*=.01). As shown in Figure 1, the number of active QardioArm users per week (defined as use at least once a week) in both the S+Q and Q groups decreased over time but was higher in the S+Q group (4.8 vs 2.4 users). Due to the small sample size and the risk of overinterpretation, statistical analysis is not included. In the Q group, the number of intervention days used by each active participant was 48, 14, 4, and 2 for each of the 4 active participants. The other 5 participants did not record QardioArm use. In the S+Q group, the number of intervention days used by each active participant was 57, 36, 19, 17, 3, and 2 for the 6 active participants. The other 3 participants did not record QardioArm use.

Participants were also asked about their perception of their BP control, which was elicited by querying how many days of the week they felt their BP was well controlled. *Well controlled* was intentionally left to the participant’s interpretation to avoid introducing bias, given the variability in the health literacy of the participants. *Number of days of the week* was categorized as *all days of the week* for 7 days, *most days of the week* for 5 to 6 days, *half the days of the week* for 3 to 4 days, few days of the week for 1 to 2 days, and *no days of the week* for 0 days. Participants were also given the option to respond, “I’m not sure.” As shown in Multimedia Appendix 1, 7 of 9 participants in the S+Q group reported their BP to be well controlled most days of the week after the intervention compared with those in the Q (3/9) and S (2/9) groups.

Participants’ perceptions of the program, both the SHCs and QardioArm devices, varied across the 3 groups qualitatively. Participants in the S group reported less satisfaction with the SHCs compared with those in the S+Q group (4/9 vs 7/9 participants rated their SHC as *satisfactory*). The Q group expressed more frustration with their QardioArm and overall had a higher cessation rate of QardioArm use compared with the S+Q group. Although the S+Q group expressed the same challenges as the Q group with QardioArm, they were more engaged. For example, the S+Q group participants identified solutions to technical difficulties with the devices. Survey responses from this group also reflected the synergy between the QardioArm and SHC. Patients in the S+Q group reported:

The results are really inconsistent—I don’t know if I trust it or not. I am thinking of getting my own machine, though. Nobody should be on blood pressure medication if they don’t need to be so we should have a machine that works.

P12

Sometimes it works and sometimes it doesn’t, but I still use it. My health coach was very involved, he was very good, would call me every week, and I would text him when it wasn’t working and he was able to help me use it and he would see if it started working later.

P20

[SHC name] was incredibly supportive and as a result of the accountability, I was actually able to improve my diet...and has taught me how to better manage my blood pressure.

P6

In comparison, surveys returned from the Q group included:

I was excited at first, but then it just went downhill. I got tired of messing with it.”

P28

Sometimes doesn’t work properly. Had difficulty putting it on.

P3

BP values were obtained in the clinic during the first participant meeting. Each QardioArm distributed was calibrated with a clinic sphygmomanometer. Any QardioArm device that did not reliably yield a result within 5 mm Hg of the clinic sphygmomanometer was not used. Baseline values were obtained in 78% (7/9) of the participants in the S group, 56% (5/9) of the participants in the Q group, and 78% (7/9) of the participants in the S+Q group. Baseline BP values were obtained only for participants who were able to attend the first meeting with their SHC or, for Q group participants, if they were able to return during the first week of the intervention. This was done to avoid potential bias introduced by the intervention or an uncalibrated QardioArm cuff. The follow-up BPs in the S+Q and Q groups were the final readings during the fifth week of the intervention from these calibrated QardioArm cuffs. These values represented 67% (6/9) of the participants assigned to the S+Q and Q groups. The S group was asked to come into the clinic to measure their BP. Only 44% (4/9) participants in this group returned to the clinic for a follow-up BP check. Given the small sample size in this pilot study, BPs reported should be interpreted with caution. At baseline, the mean BP was 149/85 (SD 15/10) mm Hg, 142/84 (SD 9/13) mm Hg, and 139/79 (SD 14/6) mm Hg in the Q, S, and S+Q groups, respectively. At the end of the intervention, the mean BP was 145/84 (SD 9/18) mm Hg, 150/85 (SD 18/12) mm Hg, and 128/69 (SD 20/14) mm Hg in the Q, S, and S+Q groups, respectively.

## Discussion

### Principal Findings

This proof-of-concept study showed that high school students are capable of learning health coaching skills that effectively facilitate patients’ use of digital home monitoring devices to improve BP compared with either SHC or device alone. We found that it is feasible to train SHCs, as demonstrated by (1) 100% of training completion and (2) successful completion of at least one patient meeting where lifestyle modification plans were created and approved by the study coordinator. It is remarkable that even in this short 5-week intervention, 1 hypertensive emergency was detected by the QardioArm and the SHC was capable of properly referring the participant for emergent care. Participants in the S+Q group used their QardioArm more frequently across all weeks of the intervention. Compared with the Q and S groups, more participants in the S+Q group subjectively felt their BP was *well controlled* after the intervention. Thus, although this was reflective of participants’ subjective experiences, the large increase in participants reporting their BP to be well controlled after the intervention suggests that the S+Q group had a larger impact from the intervention than the other two groups. The qualitative survey data also support these results. The S+Q group was more engaged with both the Q and SHC groups than the other groups. This was apparent in their willingness to troubleshoot obstacles, use their support systems, and recognize their self-agency over managing their hypertension. In addition, multiple participants in this group reached out to the study coordinator after the completion of the 5-week trial hoping to continue to meet with their SHC. Many reported that they felt responsible for cultivating the “future doctors of Stockton.” In turn, SHCs felt accountable for their patients. In essence, they were the primary care providers for their patients, recognizing the complications of hypertension and encouraging participants to make lifestyle changes. Although the pilot study was not designed to detect a statistically significant difference in BP, the S+Q group showed a trend toward a clinically meaningful decrease in BP at the end of 5 weeks compared with the other groups. The SHC facilitated the use of the QardioArm to improve patient engagement in the S+Q group. This was reflected in both the frequency of active QardioArm use in this group as well as patient engagement in more regular BP monitoring despite challenges associated with the QardioArm.

### Future Work

This study is promising because it highlights that although the use of smartphones is prevalent, the use of self-monitoring Bluetooth-enabled devices, such as QardioArm, is widely adopted by patients in MUAs who may have low health literacy. However, the assistance of a health coach can facilitate patient engagement in lifestyle changes related to hypertension, even when the health coach is a high school student without formal training or an advanced health degree. Health literacy in this study was measured by both patient use of QardioArm and participant responses of BP control most days of the week. Participants in the S+Q group had a higher frequency of use per week compared with the Q group, and overall, they had a higher proportion of participants reporting well-controlled BP on most days of the week. This demonstrates that the participants in this group had the information they needed after the intervention to assess their health status, as it relates to their BP. This, in turn, was associated with higher rates of participant engagement.

The participants in this study were recruited from a clinic in South Stockton, a particularly underserved area in San Joaquin County, California, with a human development index score of 2.86 compared with the California average of 5.39 [21]. According to the community needs assessment conducted by the San Joaquin Department of Public Health in 2016, approximately 25% of people in this geographic region fall below the poverty line, and 1 in 10 workers cannot find employment [21]. In addition, the percentage of the population in San Joaquin County, California, insured by MediCal is 30.9%, similar to the reported MediCal and Medicare insurance coverage of the participants in this study. Hypertension is a prevalent and morbid condition affecting members of this community. For example, in the zip code 95202 which is located in south Stockton, San Joaquin County, California, there were 1749 visits and 722.5 hospitalizations related to hypertension in 2016. This is compared to 365.5 visits and 381 hospitalizations related to hypertension on average for the state of California [12]. Although we were unable to collect data on the other comorbidities of the participants because of inconsistent clinical documentation at the underresourced, rural clinic, we can use the community-level data to extrapolate the risk of serious hypertension-related outcomes to the patients studied. Therefore, this patient population is suitable for the assessment of the study interventions. Although the average initial diastolic BP was close to the goal, several studies have shown that isolated systolic hypertension is comparable with systolic-diastolic hypertension for the risk of incident heart failure and cardiovascular mortality [22]. In another study published in the *New England Journal of Medicine*, the authors showed that systolic hypertension had a greater effect on the composite outcome of myocardial infarction, hemorrhagic stroke, and ischemic stroke [23]. The study population clearly showed elevated systolic pressures in all groups at baseline, which showed a trend toward improvement in the S+Q group.

The students selected as SHCs were also from a charter school located in South Stockton, San Joaquin County, California, where 1 in 4 students dropped out of high school. This is twice the average dropout rate for the state of California. Demographic data were not collected on the SHCs, as this was not part of the IRB approval. However, all SHCs expressed a desire to go into the health field and were seeking mentorship that was not readily available within their school district.

#### Conclusions

This pilot study was designed as a proof-of-concept trial to explore the feasibility of a community-based, cost-effective integration of health coaching and digital home monitoring devices. There were several limitations to this study. Participation dropped off in week 4 of the intervention because of obstacles related to transportation and the start of the new school year for the students. However, one of the important aims of this project was to carry out the interventions in a low-resource setting with limited resources. We accepted donated QardioArm devices, and students’ participation was completely voluntary. The results are promising in that they suggest that the need for health coaching in MUAs can be met by training and engaging motivated young people, who are looking for opportunities to work with patients. It would be worthwhile to explore health coaching as a possible avenue for both improving the health of communities and fostering positive youth development. Future exploration of this cost-effective, community-engaged approach is warranted.

## Appendix

Multimedia Appendix 1 Number of participants who answered that their blood pressure was well controlled on at least 5 days per week. Q: QardioArm alone; S: student health coach alone; S+Q: student+QardioArm.

## Abbreviations

BP: blood pressure
COPD: chronic obstructive pulmonary disease
HeH: Health eHeart
IRB: institutional review board
mHealth: mobile health
MUA: medically underserved area
PCP: primary care physician
Q: QardioArm alone
S: student health coach alone
SES: socioeconomic status
SHC: student health coach
SMART goal: specific, measurable, achievable, realistic, and time-based goal
UCSF: University of California, San Francisco
YCE: youth civic engagement

## References

[1] Fryar C, Ostchega Y, Hales CM, Zhang G, Kruszon-Moran D (2017). Hypertension prevalence and control among adults: United States, 2015-2016. NCHS Data Brief.

[2] Whelton P, Carey R, Aronow W, Casey DE, Collins KJ, Himmelfarb CD, DePalma SM, Gidding S, Jamerson KA, Jones DW, MacLaughlin EJ, Muntner P, Ovbiagele B, Smith SC, Spencer CC, Stafford RS, Taler SJ, Thomas RJ, Williams KA, Williamson JD, Wright JT (2018). 2017 ACC/AHA/AAPA/ABC/ACPM/AGS/APhA/ASH/ASPC/NMA/PCNA Guideline for the prevention, detection, evaluation, and management of high blood pressure in adults: a report of the American college of cardiology/American heart association task force on clinical practice guidelines. Hypertension.

[3] Benjamin EJ, Blaha MJ, Chiuve SE, Cushman M, Das SR, Deo R, de Ferranti SD, Floyd J, Fornage M, Gillespie C, Isasi CR, Jiménez MC, Jordan LC, Judd SE, Lackland D, Lichtman JH, Lisabeth L, Liu S, Longenecker CT, Mackey RH, Matsushita K, Mozaffarian D, Mussolino ME, Nasir K, Neumar RW, Palaniappan L, Pandey DK, Thiagarajan RR, Reeves MJ, Ritchey M, Rodriguez CJ, Roth GA, Rosamond WD, Sasson C, Towfighi A, Tsao CW, Turner MB, Virani SS, Voeks JH, Willey JZ, Wilkins JT, Wu JH, Alger HM, Wong SS, Muntner P, American Heart Association Statistics Committee and Stroke Statistics Subcommittee (2017). Heart disease and stroke statistics-2017 update: a report from the American Heart Association. Circulation.

[4] Leng B, Jin Y, Li G, Chen L, Jin N (2015). Socioeconomic status and hypertension: a meta-analysis. J Hypertens.

[5] McDoom MM, Palta P, Vart P, Juraschek SP, Kucharska-Newton A, Diez Roux AV, Coresh J (2018). Late life socioeconomic status and hypertension in an aging cohort: the atherosclerosis risk in communities study. J Hypertens.

[6] Logan AG, Irvine MJ, McIsaac WJ, Tisler A, Rossos PG, Easty A, Feig DS, Cafazzo JA (2012). Effect of home blood pressure telemonitoring with self-care support on uncontrolled systolic hypertension in diabetics. Hypertension.

[7] Gagnon M, Ngangue P, Payne-Gagnon J, Desmartis M (2015). m-Health adoption by healthcare professionals: a systematic review. J Am Med Inform Assoc.

[8] Kim JY, Wineinger NE, Steinhubl SR (2016). The influence of wireless self-monitoring program on the relationship between patient activation and health behaviors, medication adherence, and blood pressure levels in hypertensive patients: a substudy of a randomized controlled trial. J Med Internet Res.

[9] Fletcher BR, Hartmann-Boyce J, Hinton L, McManus RJ (2015). The effect of self-monitoring of blood pressure on medication adherence and lifestyle factors: a systematic review and meta-analysis. Am J Hypertens.

[10] Saleh S, Farah A, Dimassi H, El Arnaout N, Constantin J, Osman M, El Morr C, Alameddine M (2018). Using mobile health to enhance outcomes of noncommunicable diseases care in rural settings and refugee camps: randomized controlled trial. JMIR Mhealth Uhealth.

[11] Patel A, Praveen D, Maharani A, Oceandy D, Pilard Q, Kohli MP, Sujarwoto S, Tampubolon G (2019). Association of multifaceted mobile technology-enabled primary care intervention with cardiovascular disease risk management in rural Indonesia. JAMA Cardiol.

[12] (2013). A!Community!Health!Needs!Assessment. Healthier San Joaquin County.

[13] Paasche-Orlow MK, Wolf MS (2010). Promoting health literacy research to reduce health disparities. J Health Commun.

[14] Mackert M, Mabry-Flynn A, Champlin S, Donovan EE, Pounders K (2016). Health literacy and health information technology adoption: the potential for a new digital divide. J Med Internet Res.

[15] Margolius D, Bodenheimer T, Bennett H, Wong J, Ngo V, Padilla G, Thom DH (2012). Health coaching to improve hypertension treatment in a low-income, minority population. Ann Fam Med.

[16] Benzo R, Vickers K, Novotny PJ, Tucker S, Hoult J, Neuenfeldt P, Connett J, Lorig K, McEvoy C (2016). Health coaching and chronic obstructive pulmonary disease rehospitalization. A randomized study. Am J Respir Crit Care Med.

[17] Chang H, Hawley N, Kalyesubula R, Siddharthan T, Checkley W, Knauf F, Rabin T (2019). Challenges to hypertension and diabetes management in rural Uganda: a qualitative study with patients, village health team members, and health care professionals. Int J Equity Health.

[18] Ballard PJ, Syme SL (2016). Engaging youth in communities: a framework for promoting adolescent and community health. J Epidemiol Community Health.

[19] Karsenti T, Fievez A (2013). The Ipad in Education: Uses, Benefits and Challenges. A Survey of 6057 Students and 302 Teachers in Quebec, Canada. CRIFPE.

[20] Thom DH, Ghorob A, Hessler D, de Vore D, Chen E, Bodenheimer TA (2013). Impact of peer health coaching on glycemic control in low-income patients with diabetes: a randomized controlled trial. Ann Fam Med.

[21] (2016). San Joaquin County 2016 Community Health Needs Assessment. Healthier San Joaquin County.

[22] Tsimploulis A, Sheriff HM, Lam PH, Dooley DJ, Anker MS, Papademetriou V, Fletcher RD, Faselis C, Fonarow GC, Deedwania P, White M, Valentova M, Blackman MR, Banach M, Morgan CJ, Alagiakrishnan K, Allman RM, Aronow WS, Anker SD, Ahmed A (2017). Corrigendum to 'systolic-diastolic hypertension versus isolated systolic hypertension and incident heart failure in older adults: insights from the cardiovascular health study' [int j cardiol 235 (2017) 11-16]. Int J Cardiol.

[23] Flint AC, Conell C, Ren X, Banki NM, Chan SL, Rao VA, Melles RB, Bhatt DL (2019). Effect of systolic and diastolic blood pressure on cardiovascular outcomes. N Engl J Med.

