# Supplementary figures and images for: Recruiting Student Health Coaches to Improve Digital Blood Pressure Management: Randomized Controlled Pilot Study
Source: JMIR Form Res. 2020 Aug 25;4(8):e13637. doi: 10.2196/13637 (PMC7479581; doi:10.2196/13637)

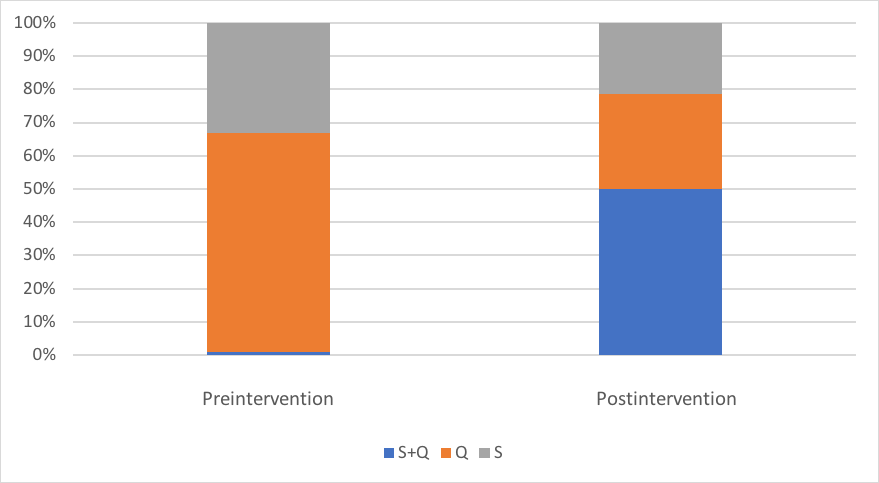

Supplement: Multimedia Appendix 1 [file formative_v4i8e13637_app1.png]
